# Family Nursing Care during the Transition to Parenthood: A Scoping Review

**DOI:** 10.3390/healthcare12050515

**Published:** 2024-02-21

**Authors:** Bruna César-Santos, Fernanda Bastos, António Dias, Maria Joana Campos

**Affiliations:** 1 Porto Nursing School, Rua Dr. Bernardino de Almeida, 4200-072 Porto, Portugal; fbastos@esenf.pt (F.B.); joana@esenf.pt (M.J.C.); 2 Saúde no Futuro Family Health Unit, Rua Bartolomeu Dias 316, 4400-043 Vila Nova de Gaia, Portugal; aacsdias@arsnorte.min-saude.pt

**Keywords:** family, family nursing, parenting, pregnancy, transition

## Abstract

Background: Family-centered care places the family at the core of care, with family nurses playing a pivotal role in supporting and guiding members through pregnancy and the transition to parenthood and acknowledging the significant adjustments during these phases. Aim: To map the evidence concerning family nurses’ care for families during the transition to parenthood. Method: The scoping review followed the Joanna Briggs Institute (JBI) methodology, focusing on family-centered care during pregnancy adaptation and the initial months of parenthood. Using a PCC (population, concept, and context) strategy, the research covered various databases: Scopus; Web of Science; and CINAHL Complete, MedLine Complete, and MedicLatina by EBSCOhost. Results: A Preferred Reporting Items for Systematic Reviews and Meta-analyses extension for Scoping Reviews (PRISMA-ScR) flow diagram was used to present the results. Eighteen articles were included, mainly from the Middle East and Europe, including cross-sectional studies and reviews. Key findings addressed the transitioning process to parenthood, the impact of family characteristics, and the role of family nurses in enhancing these processes. Conclusion: Home care is considered vital during this transition. Family nursing should concentrate on both individuals and the parental subsystem, addressing social determinants equitably. Through these efforts, they empower families to establish an optimal environment for children’s development.

## 1. Introduction

The concept of family has been evolving over time. There is no agreement on a universally accepted definition of family, and in modern times, its traditional understanding is being questioned and undergoing significant changes [1]. Nonetheless, a family can be described as a unit composed of individuals connected by blood ties, kinship, and emotional or legal relationships, comprising a system that is greater than the sum of its parts [2].

Throughout the life cycle, families experience various crises and challenges that profoundly impact the entire family. Therefore, the main family processes mediate the adaptation for all members individually, as well as their relationships and the entire family system [3]. Families are structured by individuals who share roles and responsibilities, such as caring for new family members, developing resources, and making decisions that affect everyone’s health and interactions, allowing the family to self-regulate [4]. As a system, a family can resist and recover from stressful challenges, emerging from them stronger and more resourceful. Thus, family nursing care must support the family’s efforts and encourage its members to maximize their potential during transitional periods [5].

### 1.1. Families in Transition to Parenthood

A transition is a movement or progression from one state, condition, or place to another, suggesting the eventual attainment of greater stability [6]. Nursing has played a significant role in elucidating this concept as it can influence individuals’ lives, health, and overall well-being [7]. In the specific context of the developmental transition into parenthood, aside from the inherent risks typically associated with any life transition, there exists the potential for compromising the quality of the parent–child interaction and relationship [8].

Pregnancy, a significant stage in the transition to parenthood, constitutes a complex and challenging event from a psycho-emotional perspective [8], impacting not only women but also couples and families as a whole system [9]. It encompasses what is often perceived as a joyful period in many families’ lives, yet it can equally be a cause of substantial stress due to the array of psychological, social, emotional, and physical changes [10]. The stressors associated with pregnancy include changes in body shape, the level of physical activity during this stage, and concerns related to maternal and child health and safety [11]. Additionally, unplanned pregnancy, exposure to stressful life events, and caregiving for a family member facing health challenges may contribute to additional anxiety [12]. Pregnant women may also face stressors like discrimination, financial strains, and job-related stress [12]. As the delivery date approaches, the anxiety tends to intensify [8], related to the heightened awareness of parental roles and the responsibilities in child care and marriage. Additionally, the transition to parenthood comprises the integration of new elements, modifying the dynamics and characteristics of the existing family system [13]. Thus, there are noticeable shifts in family relationships [11], along with the definition of new roles and responsibilities [13]. These aspects become focal points of attention for family nurses, underscoring the importance of addressing these evolving processes within their roles.

### 1.2. Family Nursing and the Transition to Parenthood

Family nursing involves evidence-based practices and research for family health assessment, diagnostics, interventions, and care, using contributions from different nursing models to assist families as a system [14]. This concept offers the necessary skills to support families [15,16]. Family nurses develop a therapeutic relationship to empower individuals and families for informed decision-making [14].

Family nursing is rooted in ontological and epistemological references regarding the family as the client. The extensive knowledge development in family nursing encompasses various models and theories, where the family is not only considered the client but also the fundamental unit of care, transcending the mere sum of individual elements [1]. For family nurses, possessing a profound understanding of the theoretical underpinnings is imperative to effectively employ optimal strategies for family assessment and intervention [1]. These theoretical perspectives underscore aspects such as the family’s structure, developmental transitions like the shift to parenthood, and the dynamics and performance of roles undertaken by its members.

Due to their proximity and care provision to families and individuals throughout their life cycle, family nurses can facilitate a more positive experience of parenthood. In the specific context of this transition, family nursing care emphasizes the interactions between the parental dyad and the child [1], recognizing the significance of this relationship for a child’s healthy development. Consequently, nurses should employ an approach that considers this process, as well as the dynamics within the broader family system [17]. Family-centered care (FCC) focuses on family [18]; however, individual needs should not be overlooked. It aims to promote health [19] and to connect families with collaborative, comprehensive, culturally relevant, and community-based networks of support [20].

The family’s home is a privileged context for family nursing care; therefore, a crucial strategy for promoting health, often associated with favorable outcomes for both children and families, is early home visiting [21]. Throughout the world, home visitors, such as family nurses, district nurses, community health nurses, and maternal and child health nurses, among others, have the opportunity to strengthen the capabilities of families [21].

Providing the most appropriate care for each family and its members presents a significant challenge for family nurses. They are tasked with identifying the unique needs and issues within the family. Through FCC practice, the relationships between parents and nurses are cultivated and characterized by mutual trust, respect, honesty, and open communication [20].

While there is growing evidence and positive empirical findings supporting the value and effectiveness of involving and assisting families, the incorporation of family nursing into routine care delivery has been slow and is still incomplete [22]. As a result, more research is needed to understand how evidence-based family nursing practices can lead to improved family health outcomes, particularly in the transition to parenthood [22,23]. A preliminary search of MEDLINE, the Cochrane Database of Systematic Reviews, and JBI Evidence Synthesis was conducted, and no current or underway systematic reviews or scoping reviews on the topic were identified. Therefore, the objective of this scoping review is to map the evidence concerning the family nurses’ care for families in the transition to parenthood.

## 2. Methods

This scoping review was conducted in accordance with the JBI methodology for scoping reviews [24]. The protocol was registered on the Open Science Framework (OSF): https://doi.org/10.17605/OSF.IO/5C3UG.

### 2.1. Review Questions

To perform this review, a PCC (population, concept, and context) strategy was employed. The use of the PCC mnemonic is advised to assist in formulating a concise and meaningful title as well as defining inclusion criteria for a scoping review [25]. It focused on the following review questions: “How do family nurses help families in transition to parenthood?”; “Can family nurses play a more significant role in the transition to parenthood?”; and “How can family nurses promote the family processes of integrating a new member into the family?”.

### 2.2. Eligibility Criteria

This review is part of a larger professional development project, aimed at assisting families during the transition to parenthood, between the third trimester of pregnancy and the integration of the new family member. Therefore, a specific time frame was established to align with the project’s scope and objectives. Thus, this scoping review included studies that specifically focused on families where women were pregnant in the third trimester and/or in which the new family member was already born.

The concept incorporated family-centered care. Articles exclusively addressing individual health needs were excluded.

The context integrated primary healthcare, home care, and the services provided by a range of nurses, including family nurses, district nurses, community health nurses, family nurse practitioners, public health nurses, and visiting nurses. Studies conducted in contexts such as hospital care, obstetrics/maternity settings, or care provided by midwives or other professionals rather than nurses were excluded.

### 2.3. Types of Sources

During the search phase, this scoping review considered both experimental and quasi-experimental study designs, including randomized and non-randomized controlled trials. Prospective and retrospective cohort studies, case-control studies, and analytical cross-sectional studies were considered for inclusion. This review also considered descriptive observational study designs. Qualitative research and designs such as phenomenology, grounded theory, and qualitative description were also considered. In addition, reviews that met the inclusion criteria were also considered, depending on the research question. Opinion papers, non-peer-reviewed articles, and those not indexed were excluded from consideration in this review.

### 2.4. Search Strategy

An initial limited search of MEDLINE and CINAHL was undertaken to identify articles, aiming to locate both published and unpublished studies. The text words contained in the titles and abstracts of the relevant articles, along with the index terms used to describe the studies, were employed to develop a comprehensive search strategy, conducted on 25 July 2023 (see Appendix A: Table A1 and Table A2). The search strategy, including all identified keywords and index terms, was adapted for each included database or information source. The reference list of all the included sources of evidence was screened for additional studies. Articles published in Portuguese, English, and Spanish, published since 2017, were included to assess the most recent evidence. The searched databases included Scopus; Web of Science; and CINAHL Complete, MedLine Complete, and MedicLatina (by EBSCOhost). Because this is a scoping review, a quality appraisal was not conducted, which is consistent with the framework proposed by the JBI methodological guidance for scoping reviews [24].

### 2.5. Study/Source of Evidence Selection

After the search, all the identified articles were collated and uploaded into Rayyan^®^ (2022 version), and duplicates were eliminated. The decision to use Rayyan lies in its capacity to streamline and expedite the screening and selection process of research articles, enhancing efficiency and collaboration among reviewers. Two independent reviewers assessed the titles and abstracts against the inclusion criteria, and a third reviewer assessed the papers without consensus between the two initial ones. Two independent reviewers thoroughly assessed the articles that met the inclusion criteria. Any disagreements that arose between the reviewers at each stage of the selection process were resolved through discussion and consulting the third reviewer.

### 2.6. Data Extraction

The data were extracted using a data extraction tool developed by the authors, encompassing specific details about the authors, studies’ purposes, methods, key findings and implications, and limitations relevant to the review questions. The draft extraction tool was modified and revised as necessary during the process of extracting data from each included evidence source (see Appendix B: Table A3).

## 3. Results

**Figure 1:**
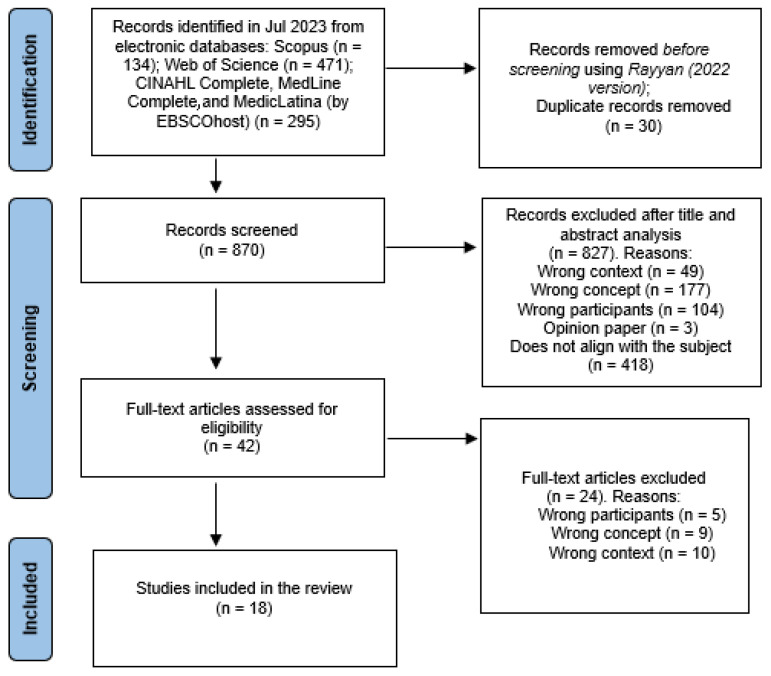
PRISMA-ScR flow diagram (adapted from [26]).

The search results and study inclusion process have been fully reported in the final scoping review and presented in a PRISMA-ScR flow diagram [26], as shown in Figure 1. Out of a total of 900 articles extracted into Rayyan^®^, 30 duplicates were removed, resulting in a final set of 870 articles for analysis. Finally, 18 articles have been included in this review. The majority of the included studies were conducted in the Middle East and Europe. The articles’ methodologies included five cross-sectional studies, five reviews, four qualitative studies, two controlled trials, one descriptive study, and one experimental study.

Table 1 comprises the salient data extracted from the included articles. For a comprehensive analysis, the complete data extraction instrument, offering detailed information, is accessible in Appendix B, Table A3.

The main findings from the included studies highlighted both the challenges and positive aspects associated with the transition to parenthood [27,28]. Furthermore, these findings have demonstrated a clear correlation with outcomes for both parents and children. One study in particular underscored the importance of recognizing men’s evolving needs during the transition to fatherhood, more than just focusing on women during this stage of life [29]. The study authors proposed a four-stage theoretical model, emphasizing the challenges and transformations fathers undergo: beginning the journey, fatherhood in limbo, facing reality, and finally settling down, where they achieve new normality and a sense of mastery.

The studies’ findings also emphasized parental well-being as a crucial factor for a positive transition to parenthood. This concept includes subjective, emotional, mental, and physical aspects, which influence the outcomes of both parents and children. Furthermore, enhanced parenting knowledge was proven to be associated with positive child development and socioemotional well-being [30]. Hence, the importance of warranting central attention to this concept in pre- and postnatal care is underscored. These findings align with the notion that it is crucial to consider various factors, such as individuals’ and families’ characteristics, and contextual elements when providing care during the transition to parenthood [27,28,31,32,33,34]. As an example, the research conducted by Nomaguchi and Milkie [27] showed that parenting challenges and well-being are influenced by social, cultural, economic, and institutional factors. It also includes birth intentions, family-friendly policies, the division of household labor, and child care. Therefore, the study underscores the importance of nurses providing families with social support at various levels and recognizing the influence of all these factors. It is important to explore parenting strains and well-being at different life stages, considering the demands and rewards of each one, and the previously mentioned contextual factors that influence parenting experiences [27].

The findings also mentioned that higher education and higher family income were related to more appropriate infant care and well-being [35]. Furthermore, working mothers and fathers with young children tend to experience less parenting stress compared to those who are not employed; however, this positive effect is influenced by the specific characteristics of one’s job [27]. Agu and colleagues’ study [31] identified being multiparous as a determinant factor for good practices. Moreover, their findings indicated that being married, older in age, residing in urban regions, and possessing a strong understanding of newborn care were additional predictors of positive practices.

In terms of economic factors, children raised in poverty and temporary shelters are at a greater risk of experiencing difficulties in language, cognitive function, and social–emotional well-being [33]. This increased risk is linked to factors such as stigma, a lack of privacy, and insecurity [28]. In less developed countries, there is a higher rate of maternal mortality associated with limited knowledge and the inadequate self-management of specific aspects of prenatal care, stemming from the lower utilization of maternal health services [36]. Regarding ethnicity, African-American mothers face increasing parenting challenges from kindergarten to third grade, unlike their White, Hispanic, Asian, and Native American counterparts [27].

The studies also underscored the vital role of family nurses in supporting families during the transition to parenthood, emphasizing the need for tailored interventions. Furthermore, they illustrated the impact of community-based primary healthcare programs on the health of both parents and children, pointing to the positive effects of perinatal education [30].

Strobel and colleagues [44] provided evidence related to FCC offered by primary healthcare services. Their study focused on various aspects, including the environment, communication, education, counseling, and support provided to families by nurses. The findings indicated that an FCC approach has a positive impact on the health and well-being of children, parents, and their families [27,33,37,38,42,44].

The articles referred to various programs and models focused on empowering parents, aiming to improve their self-efficacy and confidence, and consequently enhancing their caregiving practices [30,33,37]. For instance, the Australian Nurse-Family Partnership Program (ANFPP), based on theories of self-efficacy, attachment, and human ecology, aims to support women on their journey into motherhood, particularly those from disadvantaged backgrounds, to enhance child outcomes [37]. The assigned nurses play a pivotal role in this program, positively impacting parental abilities, pregnancy intervals, and instances of child abuse and neglect [37]. Similarly, evidence-based parenting programs like the Parent and Infant program (PIN) contribute significantly to positive child development outcomes. Emphasizing inclusive group-based services, the PIN program recognizes the importance of effective support and education during the critical first 1000 days of life, enhancing parental knowledge, skills, and confidence about their child’s development [30]. Aligned with these approaches, the Family Partnership Model (FPM) focuses on empowering parents to resolve problems through constructive feedback, home visits, day-stays, and specialized clinics. The FPM, similar to the ANFPP, highlights the significance of cultural sensitivity and underscores the role of nurses in supporting and enhancing parents’ capacity for optimal child care [42]. The model involves essential steps, including observing and recognizing the importance of cultural aspects, attributing meaning, leveraging family strengths, improving strategies for change, challenging negative self-perceptions, confronting unproductive ideas, and prioritizing parents’ self-care. These elements are considered crucial for enabling parents to provide the best possible care for their children [42]. Examining the impact of the Sit Down and Play (SDP) program, Shah and colleagues [33] found that, while there was no significant impact on parenting self-efficacy and confidence, there was a notable effect on parenting behaviors, particularly in areas related to cognitive stimulation, the provision of learning materials, and the quality of parent–child verbal interactions.

The Nurturing Care Framework (NCF), in which parents play a central role, recognizes that not all parents need the same level of support and provides guidance for policies, interventions, and services for healthcare workers to support early childhood development [45]. This framework includes strategic actions, such as home visits, parent groups, facility-based assistance, and digital helplines. It provides indicated support for children with developmental disabilities and implements activities focused on responsive caregiving and early learning. These last interventions include stimulating children’s development through routines, such as smiling, making eye contact, talking, reading and singing to them, playing with everyday household items, and others [45].

Various factors, including low social support, poor self-esteem, unpreparedness for pregnancy, family dysfunction, challenging infant temperament, and infant and maternal health issues, are connected to inadequate maternal role competence. In Dlamini and colleagues’ study, mothers who had received antenatal education exhibited better self-efficacy compared to those who had not [40], emphasizing the importance of this intervention. The studies also found a correlation between strong maternal self-efficacy and increased maternal role competency. Furthermore, this is linked with reduced postpartum depression symptoms [32,40].

Hajipour and colleagues [41] found that sleep problems during pregnancy are a significant risk factor for various adverse outcomes, like preterm birth, cesarean delivery, gestational diabetes, anemia, and low birth weight. Hence, there was an emphasis on planning suitable interventions to enhance sleep quality for better maternal outcomes. Additionally, the recognition of mental well-being impairment was underscored for early intervention [39,41].

## 4. Discussion

The discussion of the results is thematically categorized to present it more effectively: The first theme focuses on the approach to the transition to parenthood, and the second one explores families’ characteristics. The third theme examines the potential for family nurses to play a more significant role in enhancing the transition to parenthood and facilitating family adaptation to the new family member.

### 4.1. Transition to Parenthood

Parenting corresponds to the parents’ actions and responsibilities in nurturing, assisting, and guiding children as they grow and develop over their lifetime [27]. Becoming a parent involves a multifaceted, deliberate, or sometimes subconscious process of adopting specific roles [28]. Parenthood is regarded as a significant, intricate, demanding, and immensely responsible role for families and individuals [28]. As previously stated, parenthood introduces both challenges and positive aspects [10]. The former involves sustained physical, mental, and financial efforts and investments required for parenting [27]. These challenges align with the inherent changes in this transition, involving shifts in family dynamics and the roles of each member [11,12,13]. In contrast, the rewards encompass achieving parenting goals and personal growth, contributing to a positive self-concept [27].

The wide spread of intensive parenting, characterized by sensitive or responsive caregiving, has been observed across various social classes [27]. This philosophy emphasizes the critical role of a caregiver, usually the mother, in maintaining consistent engagement and emotional responsiveness and providing age-appropriate stimuli tailored to each child for optimal development. While beneficial for the child’s development, this child-centric approach demands substantial investments of parental time, financial resources, and emotional energy. Consequently, scholarly discussions have addressed the contemporary increase in parenting demands and stress, potentially resulting in decreased parental perceived life satisfaction compared to past decades [27].

Following Meleis’ Theory of Transitions [6], parenthood represents a significant life transition, marked by increased vulnerability and health-related challenges. In this context, nurses acknowledge the unique needs and changes that accompany life transitions and play a central role in helping patients and families adapt by providing knowledge and skills [28]. The individuals should internalize new knowledge, adjust their behaviors, redefine the meaning of events, and ultimately reshape their identity within their social context for a successful transition outcome [6].

Although pregnancy and the adjustment to a new child often emphasize women’s and infant’s health, the entire family is implicated in this journey, and anyone involved in a child’s care and growth contributes to the parenting process [9,28]. Fathers’ active participation in this process plays a crucial role in family settings, demonstrating a positive influence on the well-being outcomes of both mothers and children [46]. Hence, there is a need to provide new fathers with comprehensive education and support, as it fosters a sense of partnership rather than just focusing on the mother–child bond [29].

### 4.2. Families’ Characteristics

Families serve as the primary space for a child’s care, affection, reliance, and socialization. They shape habits, healthcare routines, education, and the child’s exposure to different contexts [28,33]. Serving as a context for individuals, families can function as either a positive or negative influence on an individual’s health [1]. Therefore, families’ influence spans across the child’s overall development. In the process of becoming parents, certain extended family members may play a significant role in this context. The act of sharing chores and responsibilities has been identified as a potential aid for parents in navigating the challenges of parenthood [47]. However, it may involve conditions that hinder the transition, such as inappropriate counseling and insufficient information [28]. As an example, traditional customs and teachings like the “Omugwo” practice, where mothers visit their daughters shortly after childbirth to provide care for both the baby and the mother, have been associated with limited knowledge among postnatal mothers regarding crucial aspects of newborn care, particularly breastfeeding [31]. This underscores that family support may or may not play a role in facilitating the transition. Families are not always able to provide a consistent environment and can sometimes negatively impact a child’s well-being [28].

Hence, within families, there may be both risk and protective factors that hinder a child’s development. The protective factors relate to the quality of interactions within the family and involvement in stimulating activities [28]. Furthermore, they are linked to socioeconomic factors, including family structure, parental working conditions, and support from public policies, linked with social and health determinants. On the other hand, risk factors are connected to demographic variables and socioeconomic conditions, parenting practices, and parent–child interaction styles [28]. For instance, pregnant women in less developed countries may face higher health risks [36].

Nurses, being the healthcare professionals closest to the general population, play a crucial role in health promotion and disease prevention, especially within vulnerable populations [28]. Community-based primary healthcare has the potential to enhance neonatal health and reduce mortality in rural and economically disadvantaged regions [31,38]. This context of care provides an excellent opportunity to encourage positive parenting behaviors [1], promoting a cognitively enriched home environment and sensitive parenting, as well as reducing developmental disparities linked to income [33]. Therefore, continuous healthcare assistance and education are essential for improving overall health outcomes. This includes identifying and managing risk factors for low birth weight, such as anemia, poor nutrition, hypertension, diabetes, and substance use [37]. It can be achieved through the implementation of strategies such as home visitation, which includes education on preventing complications and recognizing alarming signs. Additionally, providing early treatment or referrals for neonatal illnesses, early immunization, outreach by mobile teams, and involving participatory women’s groups can promote healthy practices during pregnancy and for newborns [37,38]. To accomplish these goals, it is essential to establish and enhance the training and deployment of community health workers [38].

In summary, there is a need for research that takes into account differences in parenting challenges and benefits based on various social contexts, life stages, and diverse countries to provide support for parents and enhance the future.

### 4.3. The Role of Family Nurses in Facilitating the Transition to Parenthood and the Adaption to the New Family Member

Proper infant care practices play a crucial role in ensuring the healthy physical and mental development of infants, and these practices are influenced by the conditions before and during childbirth, as well as the health education and health promotion delivered in the periods before and after birth [35].

Nurses play a crucial role in promoting the autonomy of parents, enhancing the quality of care, and empowering them by delivering comprehensive, continuous, and individualized care, all while valuing families’ perspectives and individualities [20,28]. Mothers ought to receive a comprehensive health education encompassing all facets of infant care practices [35]. Consequently, it is imperative to prioritize a holistic and thoughtful approach to perinatal nursing care, with a thorough assessment of individual needs, covering aspects like natural childbirth preparation, postpartum support, and breastfeeding, involving parents within their social and family context throughout the pregnancy process [28].

#### 4.3.1. Family-Centered Approaches

Promoting positive parenting behaviors is crucial to enhance children’s developmental outcomes. Recognizing family as central to health [4], several intervention programs emerged from the analysis of the included studies, essentially underscoring the importance of evidence-based and culturally sensitive interventions during critical developmental stages. They share a common thread in empowering parents, promoting positive parenting practices, and recognizing the diverse needs of families for optimal developmental outcomes.

Addressing parenting concerns and strengthening their competence is of utmost importance for healthcare promotion [48]. The stated results analyzed in this review are supported by the current evidence. Programs such as the ANFPP can yield positive results in terms of pregnancy planning and economic self-sufficiency, thereby contributing to the reduction in child maltreatment [49]. Effective home-visiting services, exemplified in programs like the FPM, play a significant role in promoting these positive outcomes by fostering family-centered home care [50]. Additionally, programs like PIN and SDP have demonstrated the potential to enhance parenting efficacy and child cognitive stimulation, particularly within the context of low-income families [48]. These improvements are anticipated to have significant implications for the present and future health outcomes of both parents and children, as well as to contribute to enhanced educational and employment prospects [51].

Upon analyzing the different programs and models, it becomes apparent that, despite their family-focused nature, the family is often perceived more as a context rather than a system in these interventions. The emphasis is placed on the parental subsystem, focusing particularly on the parent–child relationship, over other family subsystems. These interventions underscore the importance of prioritizing care within the parental unit, aiming to empower parents to effectively integrate the new family member. This approach not only promotes the health of both parents and children but also enhances the overall family process during this transitional period. Although the impact extends to the couple, the studies predominantly concentrate on the parental role.

#### 4.3.2. Well-Being Promotion

Enhancing awareness of community values and assets, and encouraging community involvement, can make early childhood development strategies more practical, efficient, and enduring [45]. A robust collaboration between community and facility-based services is vital for maintaining consistent care; therefore, healthcare providers bear the responsibility of linking families with community resources, nurturing support systems, and recognizing community leaders [45].

While parenting brings joy and meaning, it also introduces stress and strain, particularly in the early years [27], potentially leading to mental health impairment. This significantly influences how mothers engage with and care for their infants, impacting their overall ability to nurture them [40]. Mental health problems during the postpartum period can result in low maternal self-confidence, insufficient maternal caregiving skills, poor self-esteem, family dysfunction, and both infant and maternal health issues [40]. Therefore, it is crucial to prioritize comprehensive postpartum care, focusing not only on physical recovery but also on mental well-being [40]. Empowering parents to self-identify depressive symptoms is important, and programs such as parenting, psycho-education, and supportive education can play a significant role in this matter [18]. Recognizing mental illness as a family matter and consequently directing attention to the family as the primary unit of care necessitates a conceptual reorientation, potentially even prompting a paradigm shift, among healthcare professionals [52].

There is a reciprocal relationship between an individual’s health and well-being and that of their family, establishing a dynamic interdependence. When one family member falls ill, it alters the family’s daily routines and roles [1]. Partners, extended family members, and supportive friends who actively share responsibilities and provide assistance in navigating the challenges of parenthood are crucial for enhancing parental self-efficacy and competence [39,40,47]. However, the relationship between maternal role competence and postpartum depression might differ across cultures. In African settings, for instance, where discussing mental health problems is stigmatized and maternal responsibilities are often placed on women due to societal norms, postpartum depression is a risk [40]. Hence, it is imperative to consistently consider the cultural aspect of families.

Parents experience significant changes in the duration and efficiency of sleep after childbirth, especially in the initial four weeks [53]. Sleep deprivation associated with the transition to motherhood can lead to depression, which, in turn, may result in reduced bonding between mother and child, as well as family dysfunction, impacting the parents’ capacity to care for their child [39]. Hence, early intervention is seen as crucial in preventing detrimental impacts. Nurses meeting with parents should acknowledge the significance of sleep within the family and encourage parents to set aside time for themselves and to help and relieve each other, ultimately promoting and maintaining good health for the entire family [39,41]. They must address unrealistic expectations and normalize ups and downs in the parenting journey [42]. Universal parent education programs have the potential to mitigate these detrimental effects. These programs should cover diverse aspects, encompassing family dynamics; social support; infant behavior; and essential care practices, like feeding, understanding newborn sleeping patterns, maintaining hygiene, and ensuring proper immunization [54]. The need for nurses to have expertise in asking questions and creating a trusting atmosphere when addressing these concerns is also recognized [42].

Early childhood development is crucial for long-term health, well-being, learning, productivity, and nurturing care, which includes a stable and supportive environment [45]. Therefore, supporting parents and infants during the early stages of the family life cycle, through prevention and early intervention programs, is crucial for promoting optimal outcomes for parents and their children [30].

Raising children with special needs places substantial burdens on parents, especially on mothers [55]. Children facing disabilities require tailored attention [42], often requiring higher time consumption and financial costs [42,55]. Therefore, these families may experience long-term financial challenges, often accumulating unsecured debts. In addition, parents frequently face social stigma in various aspects of their lives, from interactions with medical professionals to social encounters with neighbors and friends [27]. All these factors impact families’ well-being [55].

Immunizing children also plays a vital role in fostering their overall health and well-being. The recent emergence of multiple concerns within families, attributed to various factors, may influence the uptake of vaccinations [43]. Primary care community health nurses should deliver precise and efficient details to parents to promote children’s vaccination, as the level of parental education on this matter significantly contributes to diminishing misunderstandings [43].

#### 4.3.3. Promoting Breastfeeding

The WHO recommends maintaining exclusive breastfeeding until the sixth month of the child’s age and states that infants who are not exclusively breastfed face a higher risk of mortality due to pneumonia and diarrhea compared to those who are exclusively breastfed [35,56]. Antenatal education plays a crucial role in enhancing breastfeeding knowledge and skills, and boosting the confidence needed to initiate and sustain breastfeeding up to the sixth month, ultimately influencing the baby’s health [32]. The practice of exclusive breastfeeding is influenced by improved self-efficacy among expectant mothers through antenatal nursing interventions [32]. Given the established link between increased exclusive breastfeeding, reduced infant morbidity and mortality, and improved maternal health, comprehensive antenatal education programs focusing on breastfeeding can play a crucial role in achieving better health outcomes for both infants and mothers [32]. Educational interventions that commence during the antenatal period and persist into the postpartum phase demonstrated greater effectiveness compared to approaches that solely concentrated on education during pregnancy, enhancing outcomes for both mothers and children, especially in the context of breastfeeding [34].

Addressing the review questions, the authors of this review revealed critical insights into the pivotal role of family nurses in the realms of pregnancy, the transition to parenthood, and the integration of a new member into the family. A multitude of family nursing interventions in these contexts has emerged from the literature. Home visiting was identified as one of the key intervention strategies discussed in the included studies. By visiting families at their homes, family nurses can gain an understanding of their residential building, observe the organization of home functioning, and provide on-site assistance with practical aspects of the family process.

In the context of the transition to parenthood, family nursing care focuses on empowering parents by enhancing their knowledge and capabilities in promoting their parental roles. This translates into actions like teaching, training, and assisting in specific aspects of care. It places the focus on families and their options and decisions. At the same time, through these, family nurses serve as a useful resource, helping families to identify its needs, strengths, and resources [57]. A thoughtful consideration of care provided to individuals and families is essential, transitioning from a paradigm where the family merely serves as the backdrop for care to a systemic approach.

Nurses explore the intricate landscape of the family process in adapting to this new stage of life. This involves considering the demands and rewards of each family life stage, addressing well-being aspects, and conducting thorough assessments of individual and family needs. This navigation extends beyond the individual to encompass parents within their social and family context. This review’s findings emphasize the significance of the parenting role, particularly the parent–child relationship, as family-centered care. Additionally, the examination of the results highlights the various channels through which family nurses provide assistance, underscoring their crucial role in promoting responsive parenting, early learning activities, anticipatory care, vaccination, and the effective utilization of community resources and facility-based services.

In essence, this comprehensive exploration illuminates the pivotal and multifaceted contributions of family nurses to the well-being of families during the transformative phases of pregnancy, transition to parenthood, and the integration of new members into the family unit [58].

## 5. Limitations

Some of the included articles exhibited reporting bias, lacked sample representativeness, and demonstrated potential bias toward certain geographic regions. The studies revealed a persistent gap in evidence regarding the family as a unit of care, often framing it more as a context rather than a system. Additionally, the use of the term “family nurses” may have limited access to studies referring to alternative designations. Nevertheless, the authors considered this factor in the selection of articles. The publication of studies exclusively in English could have limited the inclusivity and representation of diverse perspectives. Both of these limitations may be attributed to the varied political and healthcare services organizational landscapes across countries.The temporal restriction in the research, specifically examining studies since 2017, might have restricted a more comprehensive understanding of the subject matter. Similarly, focusing on the transition from the third trimester of pregnancy could have imposed limitations on the results.

Despite these acknowledged constraints, the authors maintain confidence in having compiled robust evidence to effectively address the study objectives and overarching research questions.

## 6. Conclusions and Implications to Practice

The transition to parenthood is significantly influenced by social determinants of health, including education, economic status, and employment, which impact family integration and individual well-being. This highlights the critical need for culturally sensitive and equity care provided by family nurses, serving as primary healthcare entry points. To deliver accurate diagnoses and tailored care, family nurses must comprehend community-specific factors, emphasizing anticipatory care and the management of parental role expectations.

Various theoretical frameworks and parenting programs underscore the significance of early support and education for positive family outcomes. It is crucial to improve the parental knowledge, skills, awareness, and understanding of the meanings associated with new roles and responsibilities. Addressing the physical, emotional, and mental well-being of parents; promoting health literacy; and offering support programs are crucial for strengthening parent–child relationships and fostering positive child development.

In clinical practice, nurses should provide guidance to families throughout parenthood, by providing holistic and integrated care, highlighting the importance of home care. Tailoring support to each family’s unique needs is essential for the effective transition to parenthood and family adaptation. Beyond the dissemination of knowledge, the analysis, discussion, confrontation, and negotiation of various aspects of parenthood are essential steps in ensuring the effectiveness and appropriateness of interventions implemented by family nurses.

Within the framework of a family-centered approach, the consistent focus on home care and community care becomes imperative. Therefore, emphasizing the significance of home visits, the formation of peer groups, and interactions with others for various activities is crucial. This family-centric model particularly underscores the dyadic relationship between the mother/father and child, which is globally sensible for nursing care, transcending cultural contexts. By prioritizing the intricacies of family subsystems and avoiding a narrow focus on couple dynamics, we ensure that nurses’ care strategies resonate universally with the diverse needs of individuals and families worldwide.

## Figures and Tables

**Table 1:** Data extraction from selected articles.

Authors and Year	Country	Methods and Participants	Main Implications for Family Nursing
Nomaguchi; Milkie 2020 [27]	Canada	Literature review	Consideration should be given to the impact of child-rearing stressors. It is crucial to recognize factors such as socioeconomic status, gender, partnership status, and race/ethnicity. Parental well-being should be a focal point in both pre- and postnatal care.
Andrade et al., 2020 [28]	Portugal	Systematic review	Families, fathers, or mothers living in adverse conditions should be considered in tailoring nursing interventions accordingly. It is crucial to address their specific needs and challenges during the care planning process.
Vidaurreta et al., 2021 [29]	Spain	Qualitative research; 14 men	The process of men’s transition to fatherhood should be considered, enabling a comprehensive understanding of their perspectives and needs at each stage of this journey.
Hickey et al., 2018 [30]	Ireland	Controlled trial; 190 parents	Creating a welcoming environment for interagency parenting support is crucial. The objective is to engage and empower parents through evidence-based prevention and early intervention.
Agu, A. et al., 2022 [31]	Nigeria	Cross-sectional study; 400 participants	It is crucial to consider the impact of community resources, families’ characteristics, and family support in the transition to parenthood.
Piro; Ahmed 2020 [32]	Iraq	Experimental investigation; 130 pregnant women	The effective provision of antenatal breastfeeding education enhances breastfeeding self-efficacy, fostering increased self-confidence, knowledge, and positive attitudes toward the practice of breastfeeding.
Shah et al., 2019 [33]	USA	Randomized controlled trial; 40 participants	Through SDP, nurses can actively promote positive parenting behaviors, such as cognitive stimulation, providing learning materials, and enhancing the quality of parent–child verbal interactions. These efforts improve developmental outcomes for children.
Herval et al., 2019 [34]	Brazil	Scoping review	Sustaining health education strategies, with a particular emphasis on improving breastfeeding practices, is one crucial approach that can be employed to enhance maternal and child outcomes.
Alobaysi; Jahan 2022 [35]	Saudi Arabia	Cross-sectional study; 200 mothers	To enhance infant care practices, it is important to address certain aspects of child care and strengthen health education initiatives for mothers.
Salih; Khalee 2022 [36]	Iraq	Descriptive cross-sectional study; 206 pregnant women	Establishing additional and consistent instructions for prenatal care within primary health centers is recommended to reduce complications during and after pregnancy.
Massi et al., 2021 [37]	Australia	Qualitative study; 76 participants	The ANFPP supports women on their journey to motherhood by providing home visits, health education, guidance, and social and emotional support. This approach aims to enhance child outcomes.
Sacks et al., 2017 [38]	USA	Systematic review	CBPHC can significantly enhance neonatal health. Key strategies include home visitation, education on preventing complications, recognizing alarming signs, providing early treatment or referrals for neonatal illnesses, early immunization, outreach by mobile teams, and participatory women’s groups.
Angelhoff et al., 2018 [39]	Sweden	Qualitative interview study; 12 parents	Sleep deprivation in parents of young children with AD should be promptly identified to prevent adverse outcomes affecting overall family well-being.
Dlamini, L. et al., 2023 [40]	Taiwan	Cross-sectional study; 343 postpartum mothers	Addressing depressive symptoms during the postpartum period is imperative to mitigate the risk of low maternal self-confidence and inadequate caregiving skills.
Hajipour et al., 2021 [41]	Iran	Multicentered cross-sectional study; 3675 pregnant women across 11 provinces	In addressing the impact of sleep quality on maternal outcomes, it is essential to plan and implement appropriate interventions within the realm of primary healthcare.
Hopwood; Clerke; Nguyen 2018 [42]	Australia	Descriptive study; 19 nurses and 60 parents from 58 different families	Through observation of various aspects and interactions of children and parents, nurses can build on the strengths of parents.
Savci et al., 2023 [43]	Turkey	Descriptive study; 193 pregnant women	Ensuring that children receive vaccinations is crucial for their overall health and well-being. Primary care community health nurses play a pivotal role in providing accurate information to parents to promote children vaccination.
Strobel et al., 2022 [44]	Australia	Systematic review	Family-centered care provided by primary healthcare services prioritizes elements such as the environment, communication, education, counseling, and support for families. It aims to improve the health and well-being of children, parents, and their families.

## Data Availability

The data that support the findings of this study are available from the corresponding authors upon reasonable request.

## Appendix A

**Table A1:** PCC strategy and MeSH terms.

Population (P)	Concept (C)	Context (C)
Pregnancy	Education	Family nursing
New family	Adaptation	Primary healthcare
Parents	Transition	
Parenthood	Family	
Newborn	Family life cycle	
	Family dynamic	
	Family relation	

**Table A2:** Search strategy.

Database	Search Terms/Boolean Phrase	Results (n)
Scopus	(TITLE-ABS-KEY (parenthood OR “new family” OR pregnancy OR parents) AND TITLE-ABS-KEY (education OR adaptation OR transition OR “family life cycle”) AND TITLE-ABS-KEY (“family nursing” OR “primary health care”) AND TITLE-ABS-KEY (“family dynamic” OR family AND relations)) AND PUBYEAR > 2016 AND PUBYEAR < 2024 AND (LIMIT-TO (DOCTYPE, “ar”) OR LIMIT-TO (DOCTYPE, “re”)) AND (LIMIT-TO (LANGUAGE, “Portuguese”) OR LIMIT-TO (LANGUAGE, “Spanish”) OR LIMIT-TO (LANGUAGE, “English”)) AND (LIMIT-TO (SRCTYPE, “j”)) AND (LIMIT-TO (PUBSTAGE, “final”))	n = 134
CINAHL Complete, MedLine Complete, and MedicLatina (by EBSCOhost)	AB (newborn OR parenthood OR pregnancy OR parents) AND AB (family nursing OR primary health care) AND AB (education Or preparation) Limiters – Scientific Magazines (Peer Reviewed); Publication Date: 20170101-20231231; Expanders: Apply to equivalent subjects; Restrict by language: Spanish; Restrict by language: Portuguese; Restrict by language: English; Search modes: Boolean/Phrase 150 duplicates in the different databases were excluded, exporting 295 articles in total	n = 295
Web of Science	Results for TI = (parenthood OR pregnancy OR parents AND education OR adaptation OR transition AND “family nursing” OR “primary health care” AND “family dynamic” OR “family relations”) and Highly Cited Papers and 2024 or 2023 or 2022 or 2021 or 2020 or 2019 or 2018 or 2017 (Publication Years) and Review Article or Article (Document Types) and Highly Cited Papers and Article or Review Article (Document Types)	n = 471
		Total n = 900

## Appendix B

**Table A3:** Total draft extraction tool.

Authors, Year, Country	Title	Purpose	Methods and Participants	Main Implications for Family Nursing	Limitations
Agu, A.P.; Akamike, I.C.; Okedo-Alex, I.N.; Umeokonkwo, A.A.; Ogbonna-Igwenyi, C.O.; Madumere, O.D; Keke, C.O. (2022) [31] Nigeria.	Predictors of knowledge and practice of newborn care among post-natal mothers attending immunization clinics in Southeast Nigeria	To assess the knowledge, practice-associated factors, and predictors of essential newborn care among post-natal mothers.	Cross-sectional study. The population included two primary healthcare centers in Southeast Nigeria, with post-natal mothers who attended immunization clinics.	Family support is crucial during the transition to parenthood. Factors like marital status, age, residency, number of previous children, and the level of knowledge about newborn care should be taken into account as potential predictors of parenting practices.	The practice was self-reported and no observation of these mothers was performed. The generalizability of the findings may be limited. Women’s responses may have been subject to recall bias.
Alobaysi, H.; Jahan, S. (2022) [35] Saudi Arabia	Infant care practices among mothers attending well-baby clinics at primary healthcare centers in Unaizah City.	To determine practices regarding infant care and to explore the association of these practices with mothers’ demographic data.	Cross-sectional study using a self-administrated questionnaires. A total of 200 mothers attending well-baby clinics in primary healthcare centers (PHCCs) in Unaizah city, Saudi Arabia.	It is important to address infant immunization, ensure timely weaning, supervise the use of formula milk in hospitals, advocate for appropriate infant sleep positions, regulate the use of pacifiers, and strengthen health education initiatives for mothers, to enhance infant care practices.	Social desirability bias cannot be ruled out. This study was carried out in a single city, restricting the applicability of the findings.
Andrade, F.M.R.; Simões Figueiredo, A.; Capelas, M.L.; Charepe, Z.; Deodato, S. (2020) [28] Portugal	Experiences of Homeless Families in Parenthood: A Systematic Review and Synthesis of Qualitative Evidence.	To identify the available data and to develop a framework to address the life experiences of homeless families in parenthood.	Systematic review, considering all the studies that focused on homeless families constituted by adult parents, over 18 years of age, and their children, under 18 years of age.	It is crucial to take into consideration families, fathers, or mothers living in adverse conditions to tailor nursing interventions accordingly.	Self-reported experienced feelings; all works were conducted in temporary or transitional shelters; studies published in other languages that were not included; limited number of databases; and possibility of having excluded other valuable text types.
Angelhoff, C.; Askenteg, H.; Wikner, U.; Edéll-Gustafsson, U. (2018) [39] Sweden	“To Cope with Everyday Life, I Need to Sleep”—A Phenomenographic Study Exploring Sleep Loss in Parents of Children with Atopic Dermatitis.	To explore and describe perceptions of sleep in parents of children under <2 years old with AD, consequences of parental sleep loss, and what strategies the parents used to manage sleep loss and to improve sleep.	Qualitative interview study of 12 parents with an inductive and descriptive design.	Nurses should promptly identify sleep deprivation in parents of young children with AD to prevent adverse outcomes affecting overall family well-being.	Small sample.
Dlamini, L.P.; Hsu, Y.Y.; Shongwe, M.C.; Wang, S.T.; Gau, M.L. (2023) [40] Taiwan	Maternal Self-Efficacy as a Mediator in the Relationship Between Postpartum Depression and Maternal Role Competence: A Cross-Sectional Survey.	To examine the relationships among postpartum depression, maternal self-efficacy, and maternal role competence.	Cross-sectional design, with 343 postpartum mothers from 3 primary healthcare facilities in Eswatini.	It is imperative to identify and address depressive symptoms during the postpartum period to mitigate the risk of low maternal self-confidence and inadequate caregiving skills.	Potential selection bias; potential recall bias. The cross-sectional nature of the study only infers an association and does not establish a cause-and-effect relationship among the studied variables.
Hajipour, M., Soltani, M., Safari-Faramani, R., Khazaei, S., Etemad, K., Rahmann, S., Valadbeigi, T., Yaghoobi, H., Rezaeian, S. (2021) [41] Iran	Maternal Sleep and Related Pregnancy Outcomes: A Multicenter Cross-Sectional Study in 11 Provinces of Iran.	To assess the association between sleep disturbance in pregnancy and maternal and child outcomes.	Multicenter cross-sectional study, conducted on 3675 pregnant women across 11 provinces in Iran in 2018.	In addressing the impact of sleep quality on maternal outcomes, it is essential to plan and implement appropriate interventions within the realm of primary healthcare.	
Herval, A.; Dumont, D.P.; Gomes, V.E.; Vargas, A.M.D.; Schaller, B. (2019) [34] Brazil	Health education strategies targeting maternal and child health: A scoping review of educational methodologies.	To identify health education strategies targeting pregnant women to improve results of pregnancy at an urban level.	Scoping review.	Various health education strategies can be employed to enhance maternal and child outcomes. One crucial approach involves sustaining health education strategies beyond childbirth, with a particular emphasis on improving breastfeeding practices.	Most of the studies included were developed in high-income countries; therefore, the results should be carefully analyzed by policy makers from low- and middle-income regions and populations. Some studies did not provide enough information about how often and for how long people were educated.
Hickey, G.; McGilloway, S.; Leckey, Y.; Stokes, A. (2018) [30] Ireland	A Universal Early Parenting Education Intervention in Community-Based Primary Care Settings: Development and Installation Challenges.	To provide an overview of the development and setting up of the Parent and Infant (PIN) program and to explore its cost-effectiveness.	Multi-method evaluation; controlled trial evaluation. Total participants: 190 parents.	Creating a welcoming environment for interagency parenting support, especially during the child’s first 1000 days, is crucial. The objective is to engage and empower parents through evidence-based prevention and early intervention.	
Hopwood, N.; Clerke, T.; Nguyen, A. (2018) [42] Australia	A pedagogical framework for facilitating parents’ learning in nurse–parent Partnership.	To examine the role of nurses in facilitating parents’ learning in services for families with young children.	Descriptive study. Observational data were collected in home visiting, day-stay, and toddler clinics in 3 Local Health Districts (LHDs). Participants: 19 nurses and 60 parents from 58 different families.	Through observation of various aspects of children and parents, as well as their interactions, nurses can facilitate the transformative process by building on the strengths of parents.	
Massi, L.; Hickey, S.; Maidment, S.J.; Roe, Y.; Kildea, S.; Nelson, C.; Kruske, S. (2021) [37] Australia	Improving interagency service integration of the Australian Nurse-Family Partnership Program for First Nations women and babies: a qualitative study.	To explore the barriers and enablers to interagency service integration for the ANFPP in an urban setting.	Qualitative study with 76 participants.	The ANFPP supports women on their journey to motherhood by providing home visits, health education, guidance, and social and emotional support, with a particular focus on those from disadvantaged backgrounds. This approach aims to enhance child outcomes.	Data from women who left the program were included in the analysis. Some participants may have not expressed their views openly. The results may not be transferable to other settings, such as regional and remote locations.
Nomaguchi, K; Milkie, M. (2020) [27] Canada	Parenthood and Well-Being: A Decade in Review.	To provide a critical review of scholarship on parenthood and well-being in advanced economies published from 2010 to 2019.	Literature review of scholarly works published as peer-reviewed journal articles, books, and book chapters from 2010 to 2019.	Consideration should be given to the impact of child-rearing stressors. It is crucial to recognize variations in these stressors based on factors such as socioeconomic status, gender, partnership status, and race/ethnicity. Parental well-being should be a focal point in both pre- and postnatal care, emphasizing the importance of providing families with multifaceted social support.	Due to space limitations, this review is highly selective, focusing on significant themes in research from the past decade.
Piro, S.S.; Ahmed, H.M. (2020) [32] Iraq	Impacts of antenatal nursing interventions on mothers’ breastfeeding self-efficacy: an experimental study.	To evaluate the role of nursing intervention on mother’s breastfeeding self-efficacy.	Experimental investigation with 130 pregnant women who attended a primary healthcare center.	Effective provision of antenatal breastfeeding education enhances breastfeeding self-efficacy, subsequently fostering increased self-confidence, knowledge, and positive attitudes toward the practice of exclusive breastfeeding.	The study sample is derived from one PHCC; so, findings generalization was not possible. Response bias might have occurred. The results may have been influenced by the personality and environment of the mother. The researcher’s preconceived expectations may have influenced their interaction with participants.
Sacks, E.; Freeman, P.A.; Sakyi, K.; Jennings, M.C.; Rassekh, B.M.; Gupta, S.; Perry, H.B. (2017) [38] USA	A comprehensive review of the evidence regarding the effectiveness of community-based primary healthcare in improving maternal, neonatal and child health: 3.Neonatal health findings.	To review the available evidence regarding the effectiveness of community-based primary healthcare (CBPHC) and common components of programs aiming to improve health during the first 28 days of life.	Systematic review of the effectiveness of projects, programs, and field research studies in improving maternal, neonatal, and child health through CBPHC.	CBPHC can significantly enhance neonatal health, particularly in settings with high mortality rates and limited resources. Key strategies include home visitation, education on preventing complications, recognizing alarming signs, providing early treatment or referrals, early immunization, outreach, and involving participatory women’s groups.	Notable geographic bias. Many of the studies were pilot studies rather than large-scale projects. A lack of information on the quality of care and the diverse definitions and measurements used in the studies.
Salih, B.B.; Khaleel, M.A. (2022) [36] Iraq	A Study on Pregnant Mothers’ Knowledge and Self-Management Toward Prenatal Care Services in Baghdad City.	To assess pregnant mothers’ knowledge and self-management toward prenatal care services by attending primary healthcare facilities in Baghdad City.	Descriptive cross-sectional study with 206 pregnant women taking prenatal care services in 3 primary healthcare centers of Baghdad City.	In order to reduce complications during and after pregnancy, it is recommended to establish additional and consistent instructions for prenatal care within primary health centers. These instructions should be presented in a clear and easily understandable format.	
Savci Bakan, A.; Aktas, B.; Yalcinoz Baysal, H.; Aykut, N. (2023) [43] Turkey	An Investigation of Pregnant Women’s Attitudes Towards Childhood Vaccination and Trust in Health Services.	To investigate pregnant women’s attitudes toward childhood vaccination and trust in health services.	Descriptive study, conducted in a city located in the eastern part of Turkey with 193 volunteer pregnant women.	Ensuring that children receive vaccinations is crucial for their overall health and well-being. Primary care community health nurses play a pivotal role in providing accurate information to parents, as parental education significantly reduces misunderstandings and promotes the vaccination of children.	
Shah, R.; Isaia, A.; Schwartz, A.; Atkins, M. (2019) [33] USA	Encouraging Parenting Behaviours that Promote Early Childhood Development Among Caregivers From Low-Income Urban Communities: A Randomized Static Group Comparison Trial of a Primary Care-Based Parenting Program.	To assess if the Sit Down and Play program can be successful in impacting key parenting behaviors that promote early childhood development.	Randomized controlled trial with an ethnically diverse group of predominantly low-income caregivers of children 2–6 months of age.	Through SDP, nurses can actively promote positive parenting behaviors, such as cognitive stimulation, providing learning materials, and enhancing the quality of parent–child verbal interactions. These efforts can contribute to improved developmental outcomes for children.	Small sample size; exclusion of families who did not speak English; recruitment from a single practice; potential recall bias; possible performance bias; and selection bias in allocation to the control versus intervention group.
Strobel, N.; Chamberlain, C.; Campbell, S.; Shields, L.; Bainbridge, R.; Adams, C.; Edmond, K.; Marriott, R.; McCalman, J. (2022) [44] Australia	Family-centered interventions for Indigenous early childhood well-being by primary healthcare services.	To evaluate the benefits and harms of family-centered interventions on a range of outcomes of Indigenous children, parents, and families.	Systematic review.	Family-centered care provided by primary healthcare services prioritizes elements such as the environment, communication, education, counseling, and support for families. Its objective is to improve the health and well-being of children, parents, and their families.	The quality of evidence for all outcomes is quite low.
Vidaurreta, M.; Lopez-Dicastillo, O.; Serrano-Monzó, I.; Belintxon, M.; Bermejo-Martins, E.; Mujika, A. (2021) [29] Spain	Placing myself in a new normalized life: The process of becoming a first-time father. A grounded theory study.	To explore the process of men becoming first-time fathers and the experiences and challenges involved.	Qualitative research to explore the experiences of 14 men during pregnancy and childbirth in different stages of pregnancy, childbirth, and the postpartum period.	Nurses should take into account the process of men’s transition to fatherhood, enabling a comprehensive understanding of their perspectives and needs at each stage of this transition.	Small sample size and the focus on men whose partners had singleton pregnancies without health risks.
